# Psychometric Properties of Prodromal Questionnaire-Brief Version among Chinese Help-Seeking Individuals

**DOI:** 10.1371/journal.pone.0148935

**Published:** 2016-02-09

**Authors:** LiHua Xu, TianHong Zhang, LiNa Zheng, HuiJun Li, YingYing Tang, XingGuang Luo, JianHua Sheng, JiJun Wang

**Affiliations:** 1 Shanghai Key Laboratory of Psychotic Disorders, Bio-X Institutes, Key Laboratory for the Genetics of Developmental and Neuropsychiatric Disorders (Ministry of Education), Shanghai Mental Health Center, Shanghai Jiaotong University School of Medicine, Shanghai, China; 2 Department of Psychiatry, Liaocheng People's Hospital, Liaocheng, Shandong, China; 3 Department of Psychology, Florida Agricultural & Mechanical University, Tallahassee, Florida, United States of America; 4 Department of Psychiatry, Beth Israel Deaconess Medical Center, Harvard Medical School, Boston, Massachusetts, United States of America; 5 Department of Psychiatry, Yale University School of Medicine, New Haven, Connecticut, United States of America; 6 VA Connecticut Healthcare System, West Haven Campus, West Haven, Connecticut, United States of America; 7 Biological Psychiatry Research Center, Beijing Huilongguan Hospital, Beijing, China; King's College London, UNITED KINGDOM

## Abstract

Prodromal Questionnaire (PQ) and Structured Interview for Prodromal Syndromes (SIPS) have been used as a two-stage process for identifying subjects at clinical high risk (CHR) of psychosis. The Prodromal Questionnaire-Brief version (PQ-B) contains 21 items derived from the PQ. The present study aimed to examine the psychometric properties of PQ-B in a Chinese help-seeking outpatient sample and to explore which items can better predict CHR diagnosis by SIPS and future transition to psychosis. In our preliminary epidemiological study, 1461 patients from a pool of 2101 individuals (15–45 years of age) completed the two-stage process. In the present study, 239 (20%) people were randomly selected among the sample who met the initial PQ-B screening criteria but had no positive diagnosis on SIPS, as well as 72 individuals with negative results on both PQ-B and SIPS, 89 prodromal and 105 psychotic subjects, yielding a total of 505 participants. The internal consistency coefficient for the PQ-B was good, with a Cronbach’s alpha of 0.897. The concordant validity of PQ-B with SIPS dichotomized diagnosis of prodrome/psychosis versus no psychosis was 0.54. To ensure 80% or a higher sensitivity and a certain specificity, 7 and 24 were respectively set as the cutoff points for the PQ-B total score and distress score for Chinese help-seeking outpatients. A logistic regression model based on six PQ-B items could allow predicting the psychotic diagnosis on SIPS, with an accuracy of 65.8%. Prodromal individuals who scored higher on the 12^th^ item of PQ-B (Do you worry at times that something may be wrong with your mind?) were less likely to convert to psychosis. PQ-B is a useful instrument for screening CHR subjects, but the cutoff score may be higher than that recommended by the author scores for help-seeking individuals in outpatient clinics. Some specific PQ-B items may have significant predictive power on dichotomized SIPS diagnoses and deserve special attention from researchers in future studies.

## Introduction

Study of psychosis risk syndromes has been based on research of early symptoms of schizophrenia[1]. Hafner et al.[2] conducted a retrospective study and investigated patients with first-episode psychosis. Their study demonstrated that 73% of patients with schizophrenia underwent a prodromal phase, which lasted 5 years on average before the development of a full psychotic disorder. During the prodromal phase[3, 4], the so-called “ultra high risk” for psychosis, most of the patients experienced an emergence of sub-clinical psychotic symptoms and gradual functional decline, which were the main reasons for patient distress. These patients suffered distress and they were aware of their mental state, and usually sought help[1, 5]. However, our previous epidemiological study[6] showed that most prodromal individuals were diagnosed with mood or anxiety disorders.

It is important to identify prodromal psychosis to prevent or postpone the onset of psychotic disorders, especially schizophrenia, and to reduce the duration of untreated psychosis, thereby improving prognosis. Instruments have been developed to identify individuals in the prodromal phase of psychosis, including self-report screening scales and clinical interviews. The former includes Prodromal Questionnaire (PQ)[7], Prime Screen[8], and Self-Screen- Prodrome[9, 10]; the latter mainly consists of Structured Interview for Prodromal Syndromes (SIPS)[11–13] and Comprehensive Assessment of At-Risk Mental States (CAARMS)[14].

We have used PQ and SIPS in a two-stage identification process[7, 15]. The PQ was initially developed by Loewy et al.[7] as a screening tool, and its first version contained 92 items, which showed moderate concurrent validity with SIPS diagnoses, 90% sensitivity and 49% specificity. To improve its efficiency and accuracy, Loewy`s team successively modified the 92-item PQ into a 21-item version[16] and a 16-item version[17], and each has been validated using SIPS and CAARMS criteria. The shorter versions had a higher specificity and an equivalent sensitivity compared to the initial version. All three versions had already been translated into Chinese[6, 18, 19]. Our research team translated and used the 21-item PQ, the Brief version (PQ-B), to screen patients at ultra-high risk of psychosis among mental health help-seeking populations.

We screened patients who came to the Shanghai Psychotherapy and Psychological Counseling Center (SPCC) to seek help for the first time, using the cutoff point for the total and distress scores (3 and 6, respectively) of the PQ-B given by Loery et al.[16]. We found that the PQ-B differentiated individuals with potentially psychotic symptoms from other help-seeking clients with an excellent sensitivity but a low specificity[6]. Many patients who got a score above the traditional PQ-B cutoff point failed to meet the criteria for prodromal psychosis based on SIPS. Therefore, the aims of the present study were: (1) to test and verify the psychometric properties of PQ-B; (2) to reset the cutoff point of PQ-B for a Chinese help-seeking outpatient sample; and more importantly, (3) to explore which PQ-B items can better predict CHR diagnosis by SIPS or even to foresee future transition to psychosis.

## Materials and Methods

### Materials

#### Prodromal Questionnaire-Brief Version (PQ-B)

The 21 item PQ-B is a self-report measure that was shown to be an effective and efficient instrument for screening psychosis risk syndromes[16]; the 21-item PQ-B is presented in the Appendix. Respondents indicate the presence or absence of each symptom item with a “Yes/No” response. The total score is the number of items marked “Yes.” For each endorsed symptom, respondents who check “Yes” are asked to answer the question “When this happens, I feel frightened, concerned, or it causes problems for me” to indicate to what extent the symptom causes distress on a 5-point Likert scale (e.g., strongly disagree, disagree, neutral, agree, strongly agree). The distress score is the sum of each item`s score on the PQ-B Likert scale.

The English version of the PQ-B was translated into Chinese with the author`s permission. The Chinese version was translated back into English and checked using the original English version to minimize any potential transcultural differences. Among subjects in our pilot study, every individual indicated that the meaning of each PQ-B item could be easily understood. The internal consistency and reliability were good. Forty six psychotropic drug naïve participants completed the PQ-B twice at an interval of two weeks. The test-retest reliability was 0.87.

#### Structured Interview for Prodromal Syndromes (SIPS)

The SIPS[13] is a semi-structured interview that can be administered by a trained clinician or researcher to preclude former or current psychotic episodes and to detect the prodromal phase of psychosis. Its principal component is the Scale of Psychosis Risk Syndromes (SOPS) for evaluating the severity of positive, negative, disorganized, and general symptoms. The core of SOPS is the scale of positive symptoms which includes unusual thought content/delusional ideas (P1), suspiciousness/concept of persecution (P2), grandiose ideas (P3), abnormal perception/hallucination (P4) and disorganized communication (P5). The identification of prodrome for psychosis is mainly based on these positive symptoms. The prodromal phase of psychosis is assessed by criteria of psychosis-risk syndromes (COPS), which defines three subtypes of prodromal symptoms: brief intermittent psychotic syndrome (BIPS), attenuated positive symptom syndrome (APSS) and genetic risk and deterioration syndrome (GRDS). Among the three subtypes, APSS is the most prevalent according to literature and our own data[6]. SIPS was translated into Chinese and it has evidenced good reliability and validity [20].

### Informed Consent

The Research Ethics Committee at the Shanghai Mental Health Centre (SMHC) approved the study protocol in 2011. All participants were given a detailed explanation of the study, including a plain language statement written in their native language. Only participants who were judged by our clinicians to be fully competent to give informed consent for participation were included. The written informed consent was obtained from all participants before being recruited for the study. All investigations were conducted according to the Declaration of Helsinki. For potential participants under age 18, written informed consent was obtained from their next of kin or legal guardians.

### Participants

Our team has been engaged in research of prodromal psychosis for several years. In 2011, we completed an epidemiological study[6], during which we investigated 2101 consecutively visiting patients (15–45 years of age), who came to These patients sought help from Shanghai Psychotherapy and Psychological Counseling Center (SPCC) of SMHC for the first time for various psychological complaints, from minor afflictive problems such as work or study issues to more severe psychotic symptoms such as hearing voices when alone or feeling of being persecuted.

In our previous study[6], 1461 individuals were screened and interviewed with the PQ-B and SIPS. According to the cutoff point recommended by the author (total score = 3 or distress score = 6)[16], 1384 individuals were positive for psychotic symptoms by the PQ-B measures; after SIPS interviews, only 87 (6.3%) individuals met the criteria for prodromal psychosis and 102 (7.4%) individuals conformed to the presence of psychotic symptoms criteria (POPS). Among the 77 individuals who were negative for psychotic symptoms on the PQ-B, 2 individuals met the criteria for prodromal psychosis and 3 for psychotic disorders based on the SIPS interview. A detailed sample flowchart was presented by Zhang et al[6].

In the present study, we sequenced the cases in order of outpatient registration number, and randomly selected 20% (239) from the participants who met the PQ-B screening positive criteria but had no diagnosis on SIPS. The extraction ratio was consistent with that of the overall PQ-B-negative individuals as described in our previous study[6]. In addition, we also included 72 PQ-B- and SIPS-negative individuals, 89 prodromal and 105 psychotic subjects, constituting a sample of 505 participants in the present study. Among the 89 prodromal subjects, 67 individuals underwent a clinical follow-up, which confirmed 16 psychosis conversions within 2 years (Table 1).

**Table 1:** Characteristics of subjects included in the present study.

Characteristics	Prodrome (n = 89)	Psychosis (n = 105)	No psychosis (n = 311)
**PQ-B-positive**	87	102	239
**PQ-B-negative**	2	3	72
**Follow-up for 2 years**	67	NI	NI
**Psychosis converters**	16	NI	NI

PQ-B: prodromal questionnaire (brief version); NI: No information.

### Statistical analysis

Analyses were conducted using SPSS Statistics 19.0. All tests were two-tailed, with p value set at 0.05.

Participants were categorized into three groups: prodrome, psychosis, and no psychosis, according to SIPS diagnoses. An independent samples t-test was conducted to compare age and PQ-B scores between males and females. A Chi-square test was conducted to explore the sex distribution, and one-way analysis of variance (one-way ANOVA) was used to compare age and PQ-B scores among the three groups.

Given that the PQ-B, as a preliminary screening instrument, is comprised of positive symptoms, it may be difficult to discriminate between prodrome and psychosis, so the two groups were pooled together to examine the psychometric properties of PQ-B. A Pearson correlation was applied to examine the relationship between PQ-B scores and dichotomized SIPS diagnoses. The Cronbach’s α was calculated to test the internal consistency of the PQ-B. To retest sensitivity and specificity and reset cutoffs of the PQ-B, a receiver operating characteristic (ROC) analysis was conducted with the diagnosis of prodrome/psychosis as the state variable. To explore which PQ-B items were likely to be more predictive of prodrome/psychosis based on SIPS and the prodrome conversion into psychosis, logistic regression analyses were conducted.

## Results

### Demographic characteristics and PQ-B scores

The demographic characteristics were presented in Table 2. There was no significant difference for the constituent ratio of sex among three groups (prodrome, psychosis, and no psychosis) (χ^2^ = 1.68, df = 2, p = 0.43). Independent sample t-test demonstrated that there was a significant difference in mean age between males and females (t = -2.289, p = 0.022), but no difference in PQ-B total score (t = -0.05, p = 0.96) or PQ-B distress score (t = -0.35, p = 0.73). One-way ANOVA indicated a significant difference among three groups with regards to age (F = 10.62, df = 2, p < 0.001), PQ-B total score (F = 103.99, df = 2, p < 0.001) and PQ-B distress score (F = 107.12, df = 2, p < 0.001). A post-hoc LSD test revealed that thenon-psychotic group was older than the prodromal (p = 0.041) and the psychotic groups (p < 0.001). Both PQ-B total score and distress score were significantly lower in the non-psychotic group than in the prodromal (p < 0.001) and psychotic groups (p < 0.001). A significant difference between the prodromal and psychotic groups was only found for PQ-B total score (p = 0.016), but not for PQ-B distress score (p = 0.074).

**Table 2:** Demographic characteristics and PQ-B total and distress scores.

Variable	Prodrome	Psychosis	No Psychosis	Total sample
**Cases**	89	105	311	505
**Male (percentage)**	44 (49.4%)	51 (48.6%)	134 (43.1%)	229 (45.3%)
**Male mean age (sd)**	24.6 (7.8)	24.3 (6.4)	26.7 (7.5)	25.7 (7.4)
**Female mean age (sd)**	27.1 (7.3)	23.7 (7.6)	28.4 (6.9)	27.3 (7.3)
**Total mean age (sd)**	25.9 (7.6)	24.0 (7.0)	27.6 (7.2)	26.6 (7.4)
**PQ-B total score (sd)**	10.6 (4.9)	12.1 (4.9)	5.7 (3.9)	7.9 (5.1)
**PQ-B distress score (sd)**	41.1 (21.7)	45.6 (21.5)	20.1 (14.6)	29.1 (21.0)

### Psychometric properties of the PQ-B

#### Reliability, validity, and correlation with SIPS

The internal consistency coefficient of the PQ-B was found to be good and Cronbach’s alpha was 0.897, which was not enhanced when any item was deleted. The correlation coefficients between each item and the total PQ-B were 0.36–0.54. To observe correlation of total and distress scores of PQ-B with classified diagnoses on SIPS, the categorical variable of dichotomized diagnoses was transformed into dummy variable (prodrome/psychosis = 1, no psychosis = 0), and a Pearson correlation analysis was performed. PQ-B total score (r = 0.536, p < 0.001) and distress score (r = 0.545, p < 0.001) were significantly correlated with dichotomized SIPS diagnoses.

#### Sensitivity, specificity, and the cutoff point

**Fig 1:**
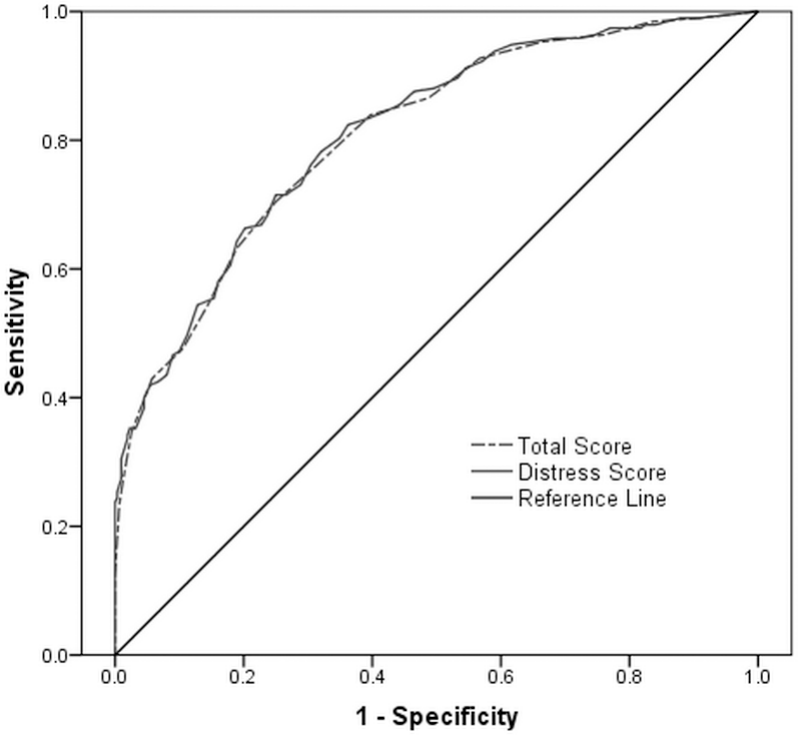
ROC curve: PQ-B total and distress score predicting prodromal/psychotic syndrome vs. no diagnosis on SIPS.

To capture the accuracy of the PQ-B and reset the cutoff point for Chinese outpatients, the receiver operating characteristic (ROC) analyses were conducted and the areas under the curve (AUCs) were calculated for the PQ-B total score and distress score, with SIPS classification as a reference standard (see Fig 1). The results showed that the PQ-B possessed significant evaluative values with 80.7% AUC [95% Confidence Interval (CI): 0.769–0.846] for the total score and 81.1% (95% CI: 0.773–0.849) for the distress score. Sensitivity and specificity at various cutoff points of the PQ-B total and distress score were presented in Table 3. Given 80% or a higher sensitivity under moderate specificity, the cutoff points of the PQ-B total score and distress score should be set at 7 and 24, respectively.

**Table 3:** Sensitivity, specificity, and AUC at various cutoff points of PQ-B scores.

PQ-B Cutoff	Sensitivity	Specificity	LR+^a^	AUC^a^	95%CI	*p*
Total score ≥ 5	92.8%	43.4%	1.64	0.81	0.77–0.85	0.000
Total score ≥ 6	86.1%	51.1%	1.76	0.81	0.77–0.85	0.000
**Total score ≥ 7**^**b**^	**83.5**%	**60.1**%	**2.09**	**0.81**	**0.77–0.85**	**0.000**
Total score ≥ 8	75.8%	68.5%	2.40	0.81	0.77–0.85	0.000
Total score ≥ 9	70.1%	74.9%	2.79	0.81	0.77–0.85	0.000
Distress score ≥ 22	84.0%	57.9%	2.00	0.81	0.77–0.85	0.000
Distress score ≥ 23	83.0%	60.5%	2.10	0.81	0.77–0.85	0.000
**Distress score ≥ 24**^**b**^	**82.0%**	**63.7%**	**2.25**	**0.81**	**0.77–0.85**	**0.000**
Distress score ≥ 25	79.9%	65.0%	2.28	0.81	0.77–0.85	0.000
Distress score ≥ 26	77.8%	67.8%	2.41	0.81	0.77–0.85	0.000

^a^ LR**+**: positive likelihood ratio; AUC: area under the curve.

^b^ cutoff point for 80% or more sensitivity under the condition of moderate specificity.

Furthermore, the sensitivity and specificity of the PQ-B among the 1461 participants who completed both PQ-B and SIPS were calculated using the cutoff point recommended by its author and the currently demarcated score, respectively. If the cutoff point of PQ-B total score was set at 3, sensitivity and specificity were 96.4% and 8.1%, respectively; at 7, they became 83.5% and 43.2%. For PQ-B distress score, if using 6 as the cutoff point, 97.4% sensitivity and 4.5% specificity were obtained; if using 24 as the cutoff point, the sensitivity remained above 80% (82.0%) but the specificity reached 46.8%.

### Predictive factors

#### Comparison of PQ-B individual item scores among three groups

**Fig 2:**
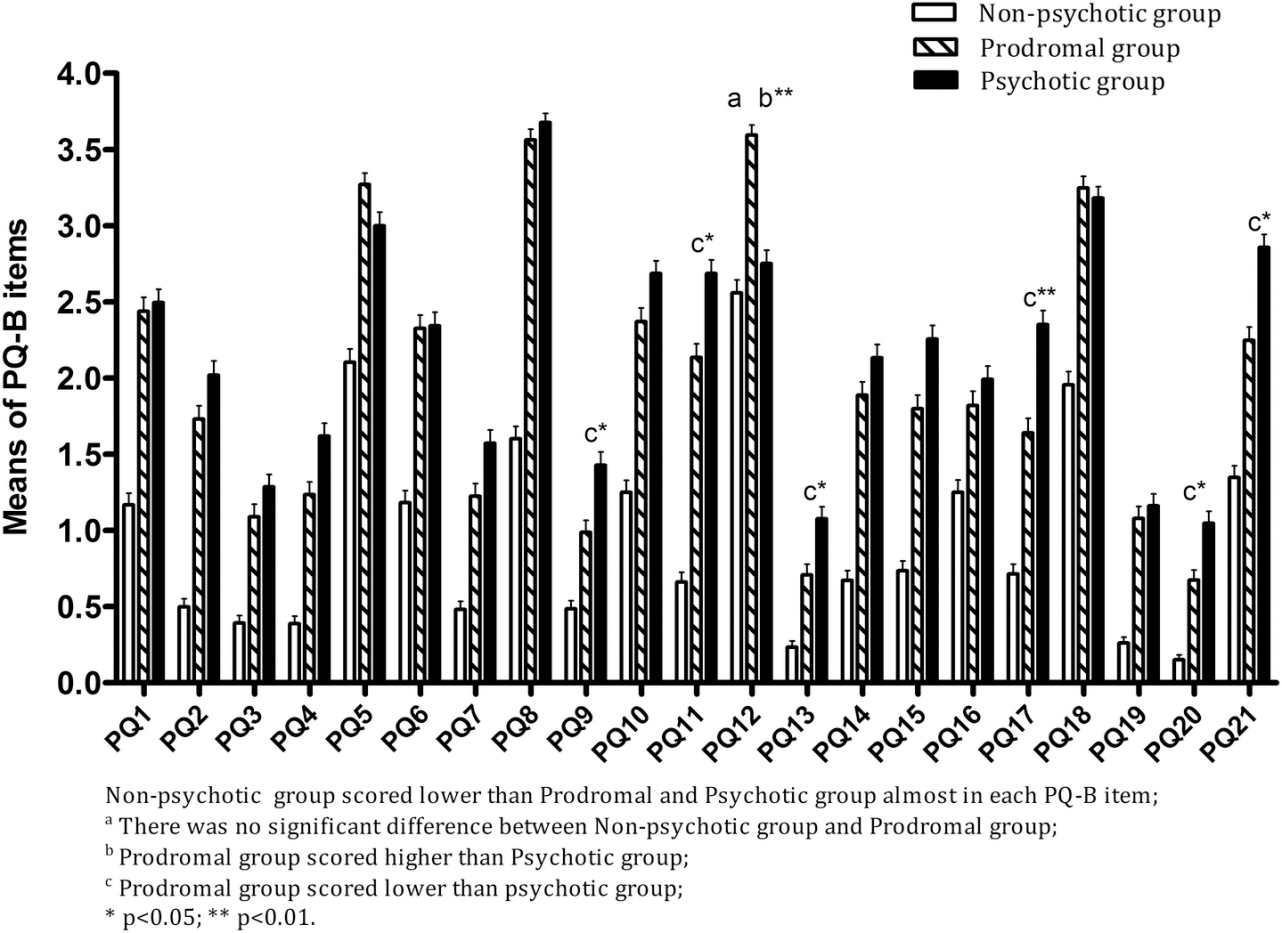
Comparison of PQ-B item scores among non-psychotic, prodromal, and psychotic groups.

To explore which items were predictive of psychosis risk syndrome, we first compared each PQ-B item score among three groups. Individual PQ-B item scores in each group were presented in a bar chart (Fig 2). One-way ANOVA revealed a significant difference among the three groups in each PQ-B item (F = 7.426–83.921, p > 0.001). Post-hoc LSD testing showed that the non-psychotic group scored lower than the prodromal and psychotic groups (p = 0.000–0.013) in each item except PQ12, which asks "Do you worry at times that something may be wrong with your mind?" Its score was significantly higher in the prodromal group (3.60 ± 1.44) than in the non-psychotic (p < 0.001 = and psychotic groups (p = 0.002), but without any difference between the latter two groups. The prodromal group scored lower than the psychotic group on PQ9 (p = 0.041), PQ11 (p = 0.023), PQ13 (p = 0.041), PQ17 (p = 0.004), PQ20 (p = 0.026), and PQ21 (p = 0.019), without any difference on other items (p = 0.067–0.949).

#### Logistic regression of SIPS dichotomized diagnoses by PQ-B items

To determine which items can predict a prodrome or psychotic diagnosis on SIPS, we set the dummy variable as mentioned above and adopted a Pearson correlation analysis. Each item was significantly correlated (r = 0.157–0.497, p < 0.001) with SIPS-classified diagnoses (prodrome/psychosis vs. no psychosis). We then employed a forward stepwise logistic regression analysis, with PQ-B item scores as independent variables and dichotomized diagnoses on SIPS as dependent variables. The results are presented in Table 4 and Table 5. Using a conditional forward stepwise method, 6 items (e.g., PQ1, PQ2, PQ4, PQ8, PQ11, and PQ14) were entered into the regression model with a statistically significant power. The percentage of correct diagnoses using this model for predicting prodrome/psychosis diagnosis was 65.8%.

**Table 4:** Logistic regression of dichotomized SIPS diagnoses by PQ-B items.

Variable	B	S.E.	Wald	Df	*p*	OR
**PQ1**	0.128	0.064	4.007	1	0.045	1.136
**PQ2**	0.269	0.076	12.525	1	0.000	1.308
**PQ4**	0.227	0.083	7.513	1	0.006	1.255
**PQ8**	0.489	0.070	48.551	1	0.000	1.631
**PQ11**	0.194	0.068	8.163	1	0.004	1.214
**PQ14**	0.197	0.072	7.456	1	0.006	1.218
**Constant**	-3.013	0.274	120.942	1	0.000	0.049
**Overall model fit test**	*χ*^2^ = 227.314, p = 0.000; Hosmer–Lemeshow test value = 12.819, p = 0.077.					
**Associated strength**	Cox-Snell R^2^ = 0.362; Nagelkerke R^2^ = 0.493					

**Table 5:** Classification table.

Observed	Predicted		Percentage Correct (%)
	No psychosis	Psychosis	
**No psychosis**	260	52	83.3
**Psychosis**	66	127	65.8

#### Psychosis transition

Among the 89 prodromal subjects, 67 (75.3%) could be reached for a two-year follow-up assessment. Sixteen (23.9%) of them converted to psychosis during this period. Among the 16 psychosis converters, 7 (43.75%) occurred within half a year, 6 (37.50%) within six to twelve months, and 3 (18.75%) after one year from the baseline SIPS interview, indicating that psychosis transition mainly happened within the first year.

Further, we analyzed the follow-up dataset (N = 67) and explored the predictive impact of PQ-B items on prodromal transition to psychosis, using a logistic regression analysis. Before that, an analysis of correlation between each PQ-B item and prognosis was conducted, in which a dummy variable for transition to psychosis was transformed to 1 and other outcomes to 0. Only PQ12 was significantly correlated with prognosis of transition (r = -0.265, p = 0.030). In addition, the correlation of PQ8 with prognosis of transition also approached a significant level (r = 0.206 and p = 0.094). A logistic regression analysis was then conducted, with items PQ8 and PQ12 as independent variables and prognosis as a dependent variable.

Although the associated strength was shown to be low (Cox-Snell R2 = 0.119, Nagelkerke R2 = 0.196), we could still obtain significant associations between PQ8 & PQ12 and transition prognosis (Omnibus test: χ^2^ = 8.513, p = 0.014; Hosmer–Lemeshow test value = 7.895, p = 0.246). Consistent with the results from the correlation analysis, the predictive effect of PQ8 on psychosis transition did not reach a significant level, while PQ12 showed a significant protective role (Wald value = 5.705, p = 0.017, OR = 0.609, regression coefficient = -0.496). The correct percentage in predicting psychosis transition was 16.7% for this model.

## Discussion

In our practical screening work for prodromal individuals in Shanghai, quite a few people were assessed as positive according to the recommended PQ-B cutoff point, but negative based on SIPS. Additionally, no other researchers reported PQ-B reliability and validity in Chinese help-seeking outpatients. In the present study, we demonstrated PQ-B psychometric properties and reset the cutoff score in a Chinese outpatient sample. We also explored which PQ-B items could better differentiate prodromal/psychotic individuals from non-psychotic ones and observed which items might play a predictive role in the prognosis of prodrome-to-psychosis transition.

Like previous studies[16, 21–23], our study also showed that PQ-B is a useful first-step instrument with good psychometric properties in the two-stage process for identifying subjects with prodromal psychosis. Among our sample, participants with prodrome or psychosis based on SIPS got significantly higher PQ-B total and distress scores than non-psychotic subjects. In addition, the PQ-B had good test-retest reliability (r = 0.87) and internal consistency coefficient, with Cronbach’s alpha equivalent to 0.897. Furthermore, it had good concurrent validity with SIPS diagnoses based on face-to-face interview, supported by its moderate evaluative value with AUC equal to 81% when distinguishing psychosis/prodrome versus no psychosis.

However, we set 7 and 24 as the cutoff points for PQ-B total score and distress score, respectively, much higher than those recommended (3 and 6, respectively)[16]. These modified cutoff points not only ensured 80% or higher sensitivity, but also improved the specificity of PQ-B total score from 8.1% to 43.2% and improved the specificity of PQ-B distress score from 4.5% to 46.8%. Although language and cultural background may contribute to the PQ-B cutoff differences between the present study and the initial validation study by Loewy et al.[16], there may be other reasons. First, the sample in the present study is from a clinical department of Shanghai Mental Health Center rather than from a community or a college. All subjects were help-seeking outpatients suffering various psychological problems, such as depression, anxiety, obsession, etc. It would be possible that this group of mixed clinical outpatients indeed experience psychosis-like symptoms which can be explained by non-psychotic disorders (i.e., patients with depression could experience that people around were thinking about them in a negative way, which would be self-reported as a positive symptom in the PQ-B test)[24–27]. They may also have cognitive deviations or dysfunctional beliefs[28–30], therefore, a false self-rating may exist. Second, all presenting first-visit outpatients were assessed in the present study, while in Loewy's study[16], all subjects were patients with some definite symptoms, who were referred to an early psychosis clinic, and therefore, it got a lower cutoff point but with a relatively higher sensitivity and specificity. Although the PQ-B has been applied in groups of prisoners, students[22, 23], and the general population[21], this is the first study testing its psychometric properties in a "pure" sample of outpatients, supporting its rational use in clinical settings. Additionally, in contrast to previous research findings[16, 23], the PQ-B distress score did not increase the sensitivity and specificity in our sample. The method of calculating distress scores may not be sensitive in screening prodromal subjects. An individual who got a high total distress score and experienced several positive symptoms but each with 2 or fewer points on the severity scale of SOPS will not meet prodromal criteria. On the other hand, another individual, who got a low total distress score and experienced only one positive symptom but with 3 or more points on the severity scale of SOPS and with a frequency of at least once every week during past month will meet the prodromal criteria. Therefore, the significance of the distress score is better to be understood as an additional description of each PQ-B item, just the severity of each symptom.

Although a considerable portion of non-psychotic participants checked "Yes" on some PQ-B items, most of them selected "Strongly disagree" or "Disagree" when they were asked to evaluate the degree of its distress "When this happens, I feel frightened, concerned, or it causes problems for me." The non-psychotic group had lower mean score on each PQ-B item in comparison with that for the prodromal and psychotic groups. However, they scored 2 or more on the 5^th^ item (PQ5; "Have you felt that you are not in control of your own ideas of thoughts?"), the 12^th^ item (PQ12; "Do you worry at times that something may be wrong with your mind?"), and the 18^th^ item (PQ18; "Do you find yourself feeling mistrustful or suspicious of other people?"). The phenomena that these 3 items refer to are likely to be present among non-psychotic patients. For example, patients with obsessive-compulsive disorder often complain that their thoughts are getting out of control[31]; patients with depression tend to have automatic negative thoughts[28, 29] and likely have no confidence in themselves and other people. Additionally, outpatients who take the initiative to come to psychotherapy and psychological counseling centers usually complain their minds are off-balance. Consistent with the aforementioned results, in the following analysis, these 3 items did not enter into the logistic regression of dichotomized SIPS diagnoses by PQ-B items.

Among all PQ-B items, the 1^st^ (PQ1), 2^nd^ (PQ2), 4^th^ (PQ4), 8^th^ (PQ8), 11^th^ (PQ11), and 14^th^ (PQ14) items entered into the logistic regression and had a significant predictive effect on dichotomized SIPS diagnoses. The correct percentage of the regression model for predicting psychosis/prodrome of SIPS was 65.8%. In general, delusional ideas, suspiciousness, and perceptual disturbances are more common among prodromal individuals than grandiose ideas and disorganized communication. In Yung and McGorry's early pilot study[3], they reported that three kinds of positive symptoms, e.g., perceptual disturbances, delusional mood, and suspiciousness, could account for 62%, 62%, and 71%, respectively, of the symptoms that prodromal individuals experienced.

Prodromal subjects got much higher score on the 12^th^ item of the PQ-B (PQ12) than psychotic and non-psychotic participants; there was no significant difference between the latter two groups. PQ12 asks "Do you worry at times that something may be wrong with your mind?” In the final analysis, we found the experience inquired by PQ12 might play a protective role in preventing prodromal patients from converting to psychosis. PQ12 examine reality-testing ability, which is one key criteria for differentiating prodrome from psychosis[1, 5]. This capacity makes prodromal individuals worry about or examine their own mental states, and further motivates them to seek help and keep well compliance to treatment.

The present study showed significant differences between prodromal and full psychotic groups in several PQ-B items (PQ9, PQ11, PQ13, PQ17, PQ20 and PQ21). However, it is still too difficult to use this subset of items to differentiate the two groups at the screening stage. First, both prodromal and fully psychotic individuals are likely to experience these positive symptom items, rather than different items, differing only in severity (high risk vs. psychotic level) or in frequency if at psychotic level (once every month vs. 4 times a week). Second, for fully psychotic patients in our sample, they could not recognize all of their positive symptoms and thus must have responded negatively on some PQ-B items in the first screening stage. It is therefore necessary to conduct a face-to-face interview to make the diagnosis.

Currently, psychiatrists in China have often paid close attention to whether patients did or did not fully display psychosis, but have had limited time and resources for identifying early signs of psychotic experiences. Therefore, a brief screening tool with a reasonable cutoff point may help them to target prodromal subjects in a relatively efficient way. Since some effective managements for prodromal patients have been suggested[32–34], it is important to integrate self-reported inquiry questions into the clinical routine to find prodromal individuals among help-seeking outpatients. Several considerations should be taken in the interpretation of our findings. First, as the sample in the present study was from a clinical setting of the largest mental health service in China, it is not known if these results can be generalized to primary care or psychiatric hospital in remote regions. Second, only a small proportion of prodromal subjects were confirmed as converters to psychosis in the limited follow-up period. It is thus possible that there were yet other undetected converters among the prodromal group; this could lead to an underestimation of the predictive power of PQ-B items. A further limitation is that we selected randomly 20% subjects with PQ-B screening positive and SIPS-negative diagnosis rather than sampling from across the scoring distribution. This could lead to a bias in the estimation of PQ-B’s sensitivity and specificity. Finally, the upper age limit of the present study (45 years) is somewhat higher than that for most psychosis risk studies; therefore, the age difference may potentially have an effect on its PQ-B scores.

## Conclusions

In conclusion, it is necessary to use a two-stage process for detecting psychosis risk syndrome in clinical settings in order to optimize the use of resources. PQ-B is a useful, self-report screening instrument with good psychometric properties in the first stage of the process. For mental health help-seeking individuals in outpatient clinics, however, the useful cutoffs of PQ-B may be higher than the recommended points. PQ-B individual items representing delusional ideas, suspiciousness, and perceptual disturbances may have significant discriminatory power to differentiate those with psychosis/prodrome from those with no psychosis. In addition, prodromal individuals who scored higher on the 12th item of PQ-B were less likely to convert to psychosis, possibly suggesting the protective role of an intact insight in preventing prodromal patients from converting to psychosis.

## Appendix

### The items on the Prodromal Questionnaire-Brief Version (PQ-B):

1. Do familiar surroundings sometimes seem strange, confusing, threatening, or unreal to you? (PQ1);
2. Have you heard unusual sounds like banging, clicking, hissing, clapping, or ringing in your ears? (PQ2);
3. Do things that you see appear different from the way they usually do (brighter or duller, larger or smaller, or changed in some other way)? (PQ3);
4. Have you had experiences with telepathy, psychic forces, or fortune telling? (PQ4);
5. Have you felt that you are not in control of your own ideas or thoughts? (PQ5);
6. Do you have difficulty getting your point across because you ramble or go off the track a lot when you talk? (PQ6);
7. Do you have strong feelings or beliefs about being unusually gifted or talented in some way? (PQ7);
8. Do you feel that other people are watching you or talking about you? (PQ8);
9. Do you sometimes get strange feelings on or just beneath your skin, like bugs crawling? (PQ9);
10. Do you sometimes feel suddenly distracted by distant sounds that you are not normally aware of? (PQ10);
11. Have you had the sense that some person or force is around you, although you couldn't see anyone? (PQ11);
12. Do you worry at times that something may be wrong with your mind? (PQ12);
13. Have you ever felt that you don't exist, the world does not exist, or that you are dead? (PQ13);
14. Have you been confused at times whether something you experienced was real or imaginary? (PQ14);
15. Do you hold beliefs that other people would find unusual or bizarre? (PQ15);
16. Do you feel that parts of your body have changed in some way, or that parts of your body are working differently? (PQ16);
17. Are your thoughts sometimes so strong that you can almost hear them? (PQ17);
18. Do you find yourself feeling mistrustful or suspicious of other people? (PQ18);
19. Have you seen unusual things like flashes, flames, blinding light, or geometric figures? (PQ19);
20. Have you seen things that other people can't see or don't seem to see? (PQ20);
21. Do people sometimes find it hard to understand what you are saying? (PQ21).

## Supporting Information

S1 File Dataset underlying the findings in the study. (XLS)
